# Supplementary material for: Where have I got to? Associations of age at marriage with marital household assets in educated and uneducated women in lowland Nepal
Source: PeerJ. 2024 Aug 7;12:e17671. doi: 10.7717/peerj.17671 (PMC11316463; doi:10.7717/peerj.17671)
Supplement: Supplemental Information 1 [file peerj-12-17671-s001.docx]

**Table S1. Bias in missing and available data on assets for analysis in women aged 12-34 years, surveyed within ≤1 years of marriage** **in lowland Nepal (*n*=3,976)**

|  | **Assets measured in natal or other household (*n*=874)** | **Assets measured in marital household**  **(*n*=3,102)** | ***p*-value^1^** |
| --- | --- | --- | --- |
|  | Median (IQR) | Median (IQR) |  |
| Women’s age (years) | 17 (2) | 17 (2) | 0.083 |
|  | Frequency (%) | Frequency (%) | ***p*-value^2^** |
| Marriage age (years) |  |  | 0.258 |
| ≤14 years | 88 (10) | 321 (10) |  |
| 15 years | 152 (17) | 598 (19) |  |
| 16 years | 248 (28) | 897 (29) |  |
| 17 years | 177 (20) | 650 (21) |  |
| ≥18 years | 209 (24) | 636 (20) |  |
|  |  |  |  |
| Women’s education level (years) |  |  | **0.003** |
| None | 397 (45) | 1223 (39) |  |
| Primary (1-5 years) | 109 (13) | 360 (12) |  |
| Lower-secondary (6-8 years) | 149 (17) | 580 (19) |  |
| Secondary or higher (≥9 years) | 219 (25) | 939 (30) |  |
|  |  |  |  |
| Husband’s education level (years) |  |  | **<0.001** |
| None | 375 (43) | 1065 (34) |  |
| Primary (1-5 years) | 76 (9) | 362 (12) |  |
| Lower-secondary (6-8 years) | 125 (14) | 576 (19) |  |
| Secondary or higher (≥9 years) | 298 (34) | 1099 (35) |  |
|  |  |  |  |
| Caste |  |  | **<0.001** |
| Disadvantaged: Muslim, Dalit | 335 (38) | 974 (31) |  |
| Middle: Janjati, other Madhesi | 329 (38) | 1371 (44) |  |
| Advantaged: Yadav, Brahmin | 210 (24) | 757 (24) |  |

*n*, number. IQR, Interquartile Range. ^1^Kruskal-Wallis test. ^2^Chi-squared test.
