# Supplementary material for: Where have I got to? Associations of age at marriage with marital household assets in educated and uneducated women in lowland Nepal
Source: PeerJ. 2024 Aug 7;12:e17671. doi: 10.7717/peerj.17671 (PMC11316463; doi:10.7717/peerj.17671)
Supplement: Supplemental Information 2 [file peerj-12-17671-s002.docx]

**Table S2. Statistical model for Figure 4, for the full sample of women aged 12-34 years, surveyed within ≤1 years of marriage (*n=*3,102)**

|  | *Dep. Var. =*  *Marital household asset score* |
| --- | --- |
|  | **Women’s marriage age, their education, and interaction terms** |
|  | *β (standard errors)* |
| Intercept | 32.9 (1.6)*** |
| Women’s marriage age groups (y)^1^ | 0.7 (0.4) |
| Women’s education (y): None | Reference |
| Primary (1-5 years) | 11.3 (3.2)*** |
| Lower-secondary (6-8 years) | 12.1 (2.5)*** |
| Secondary or higher (≥9 years) | 13.0 (2.5)*** |
| Interaction terms | Reference |
| Women’s primary education and marriage age (y) | -1.5 (0.9) |
| Women’s lower-secondary education and marriage age (y) | 0.2 (0.8) |
| Women’s secondary education and marriage age (y) | 2.1 (0.7)** |
| Marginal *R*-squared | 0.17 |
| Conditional *R*-squared | 0.24 |

*n*, number. Model includes fixed and random effects estimates for geographic clusters. ***p*<0.01, ****p*<0.001. ^1^Coded as: ≤14 years, 15 years, 16 years, 17 years and ≥18 years.
