# Supplementary material for: Where have I got to? Associations of age at marriage with marital household assets in educated and uneducated women in lowland Nepal
Source: PeerJ. 2024 Aug 7;12:e17671. doi: 10.7717/peerj.17671 (PMC11316463; doi:10.7717/peerj.17671)
Supplement: Supplemental Information 3 [file peerj-12-17671-s003.docx]

**Table S3. Quantile regression models of women’s marriage age and their education with marital household asset score for women aged 12 to 34 years, surveyed within ≤1 year of marriage (*n=*3,102)**

|  | *Dep. Var. = Marital household asset score* | | | |
| --- | --- | --- | --- | --- |
|  | **Model 1**  **Women’s marriage age** | **Model 2 Women’s education** | **Model 3**  **Women’s marriage age, their education, and interaction terms** | **Model 4**  **Women’s marriage age, their education, marital household traits and interaction terms** |
|  | *β (standard errors)* | *β (standard errors)* | *β (standard errors)* | *β (standard errors)* |
| Women’s age (y) | -0.4 (0.4) | 0.2 (0.2) | -0.5 (0.2) | -0.6 (0.3) |
| Women’s marriage age (y): ≤14y | Reference |  |  |  |
| 15 years | 1.1 (1.5) |  |  |  |
| 16 years | 3.8 (1.7)* |  |  |  |
| 17 years | 4.1 (1.7)* |  |  |  |
| ≥18 years | 7.8 (2.3)* |  |  |  |
| Women’s marriage age groups (y)^1^ for interaction models 3 and 4 |  |  | 1.0 (0.5)* | 1.4 (0.6)* |
| Women’s education (y): None |  | Reference | Reference | Reference |
| Primary (1-5 years) |  | 6.8 (0.9)*** | 10.2 (2.8)*** | 7.6 (2.9)** |
| Lower-secondary (6-8 years) |  | 12.2 (1.1)*** | 11.0 (3.1)*** | 6.5 (2.2)** |
| Secondary or higher (≥9 years) |  | 20.6 (1.2)*** | 11.9 (2.3)*** | 6.3 (2.9)* |
| Husband’s education (y): None |  |  |  | Reference |
| Primary (1-5 years) |  |  |  | 4.6 (1.4)*** |
| Lower-secondary (6-8 years) |  |  |  | 6.6 (1.1)*** |
| Secondary or higher (≥9 years) |  |  |  | 6.3 (1.0)*** |
| Caste: Disadvantaged |  |  |  | Reference |
| Middle |  |  |  | 0.3 (0.9) |
| Advantaged |  |  |  | 1.3 (1.3) |
| Interaction terms: Uneducated women |  |  | Reference | Reference |
| Women’s primary education and  marriage age (y) |  |  | -1.2 (0.8) | -1.3 (0.9) |
| Women’s lower-secondary education  and marriage age (y) |  |  | -0.03 (1.0) | 0.2 (0.7) |
| Women’s secondary education and  marriage age (y) |  |  | 2.5 (0.6)*** | 1.8 (0.9)** |

*n*, number. Models include fixed and random effects estimates for geographic clusters. **p*<0.05, ***p*<0.01, ****p*<0.001. ^1^Coded similar to Model 1: ≤14 years, 15 years, 16 years, 17 years and ≥18 years.
