# Supplementary material for: Where have I got to? Associations of age at marriage with marital household assets in educated and uneducated women in lowland Nepal
Source: PeerJ. 2024 Aug 7;12:e17671. doi: 10.7717/peerj.17671 (PMC11316463; doi:10.7717/peerj.17671)
Supplement: Supplemental Information 4 [file peerj-12-17671-s004.docx]

**Table S4. Quantile mixed-effects models of women’s marriage age with marital household asset score for only uneducated women aged 12 to 34 years, surveyed within ≤1 year of marriage (*n=*1,223)**

|  | *Dep. Var. = Marital household asset score* | |
| --- | --- | --- |
|  | **Model 1**  **Women’s marriage age** | **Model 2**  **Women’s marriage age and marital household traits** |
|  | *β (standard errors)* | *β (standard errors)* |
| Women’s age (y) | -1.1 (0.4)* | -0.9 (0.4) |
| Women’s marriage age (y): ≤14 years | Reference | Reference |
| 15 years | 2.4 (1.8) | 1.1 (1.9) |
| 16 years | 4.2 (2.0)* | 3.7 (1.7)* |
| 17 years | 3.4 (2.0) | 2.7 (2.3) |
| ≥18 years | 6.2 (3.2)* | 6.4 (2.9)* |
| Husband’s education (y): None |  | Reference |
| Primary (1-5 years) |  | 4.3 (1.6)*** |
| Lower-secondary (6-8 years) |  | 5.0 (1.3)*** |
| Secondary or higher (≥9 years) |  | 11.7 (1.9)*** |
| Caste: Disadvantaged |  | Reference |
| Middle |  | -0.1 (1.1) |
| Advantaged |  | 3.0 (1.3)* |

*n*, number. Models include fixed and random effects estimates for geographic clusters. **p*<0.05 ****p*<0.001.
