# Supplementary figures and images for: Where have I got to? Associations of age at marriage with marital household assets in educated and uneducated women in lowland Nepal
Source: PeerJ. 2024 Aug 7;12:e17671. doi: 10.7717/peerj.17671 (PMC11316463; doi:10.7717/peerj.17671)

(a)

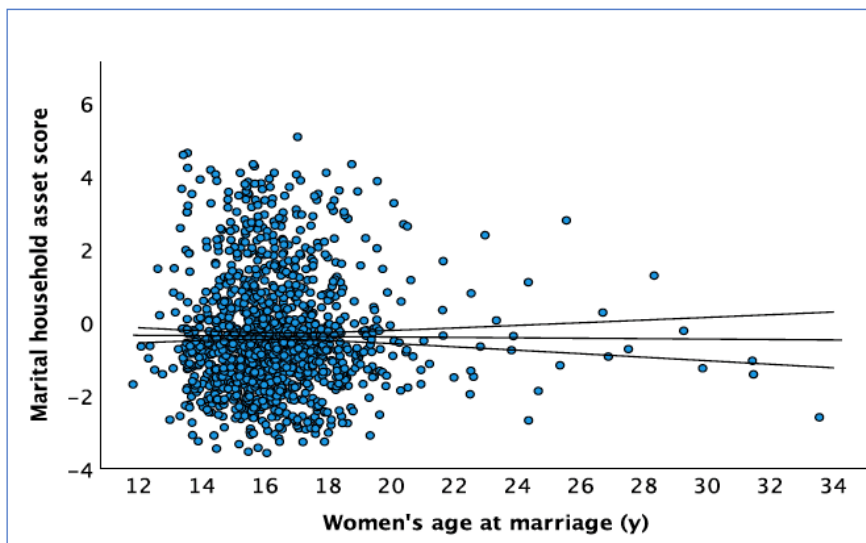

(b)

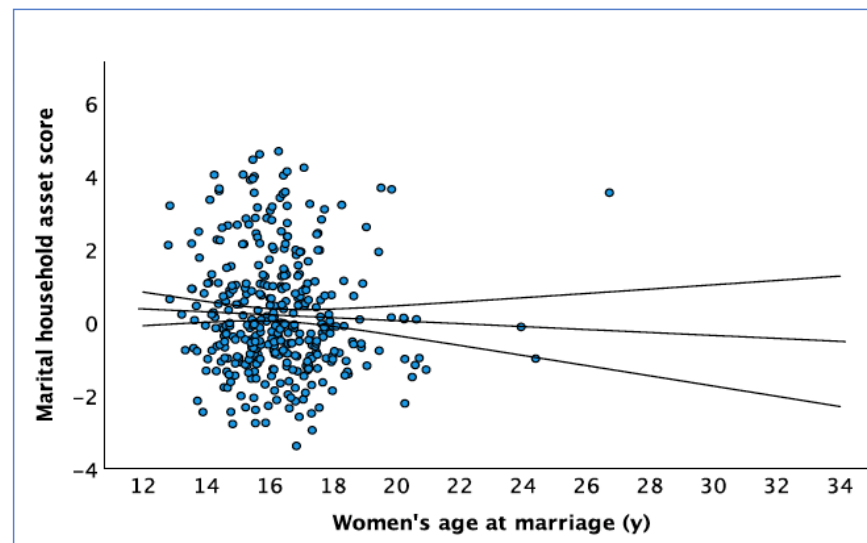

(c)

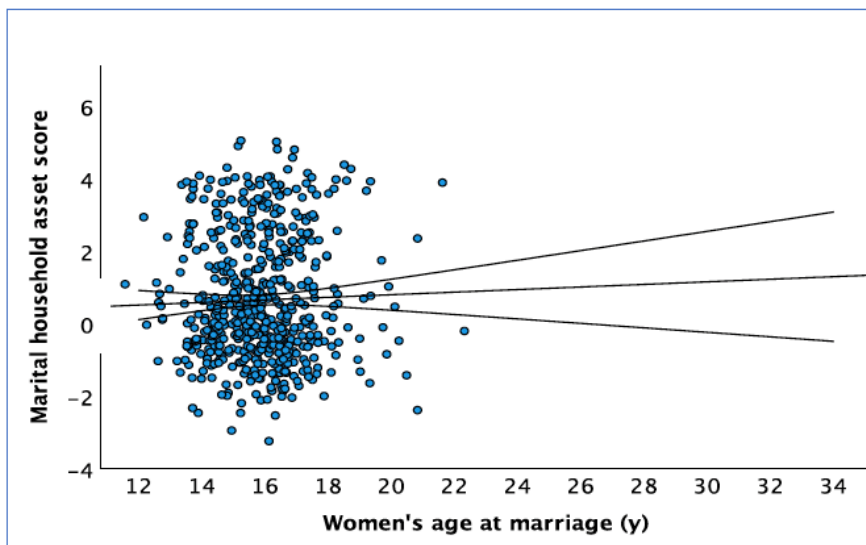

(d)

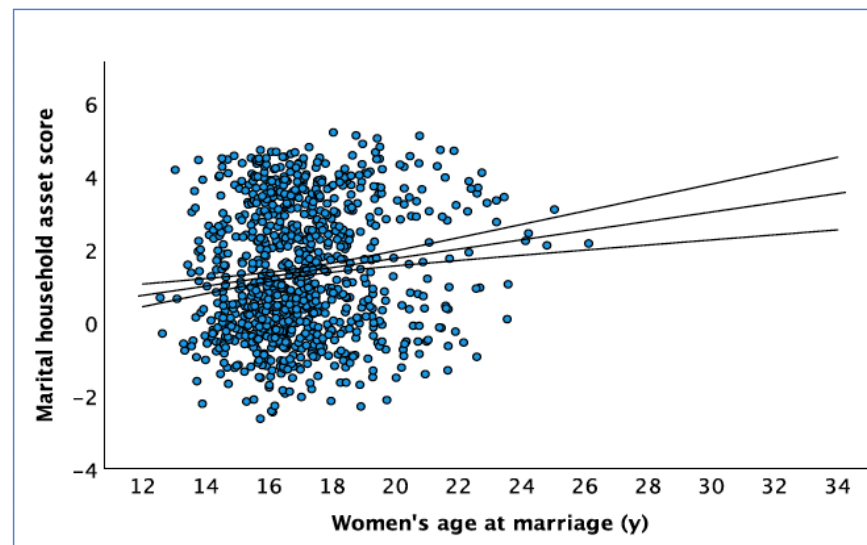

Supplement: Supplemental Information 5 — Plots use the raw values of the marital household asset score. (a) Uneducated women (n=1,223), (b) Primary education (n=360), (c) Lower-secondary education (n=580), and (d) Secondary education or higher (n=939). *Marriage age is only available in completed integer years. Jitter has been added to show more data points. Lines represent confidence intervals of the regression slope. The scale for marriage age is set from 12 to 34 years to maintain consistency across the four plots. [file peerj-12-17671-s005.pdf]

Median assets by women's education level and marriage age

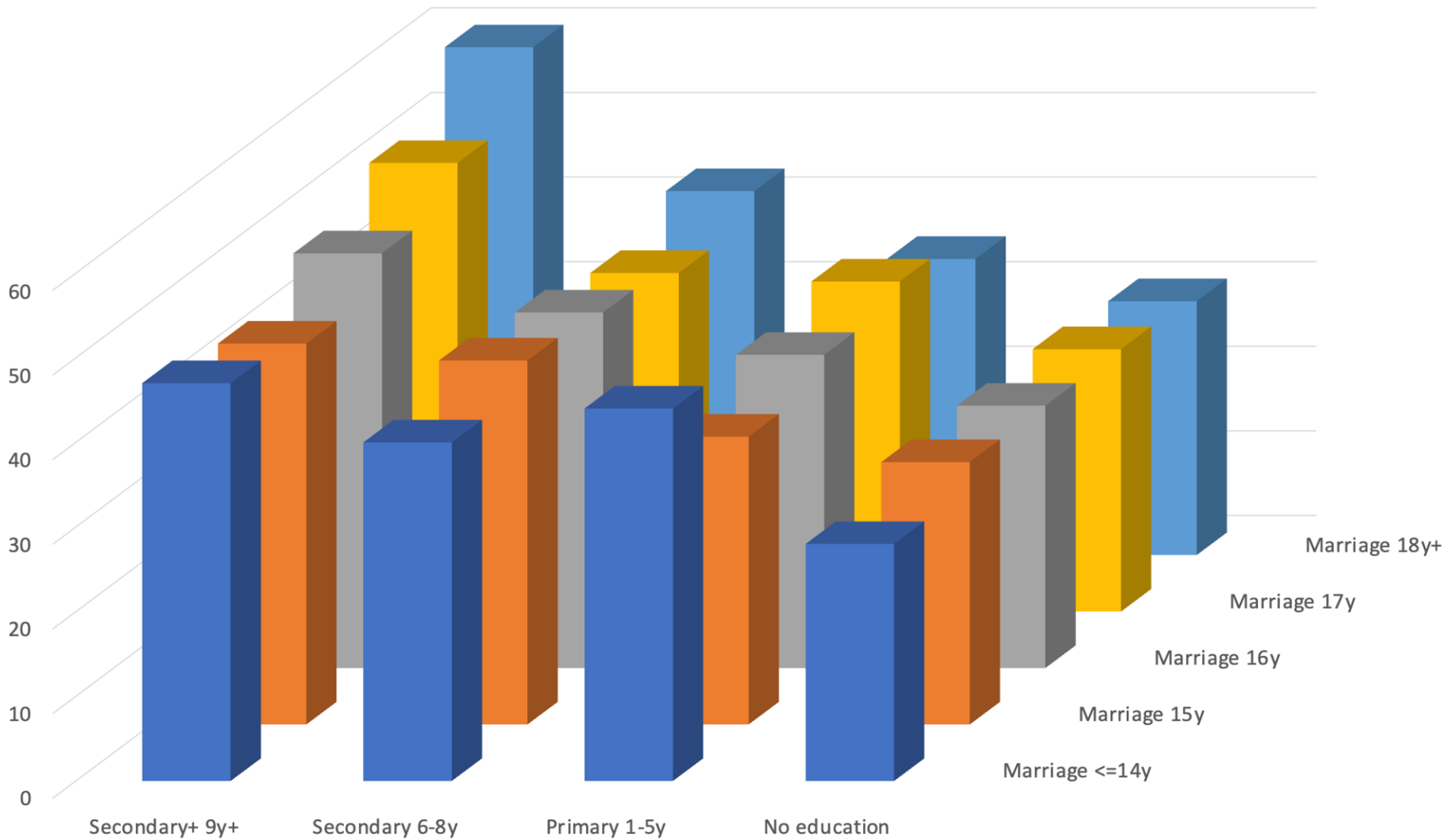

Supplement: Supplemental Information 6 — In this 3D plot, the y-axis shows marital household median asset score according to groups of women’s education level (x-axis), and groups of their age at marriage (z-axis). Among women with no education, asset score increased minimally with older age at marriage. The same pattern was evident for women with primary or lower-secondary education, though all members of these groups had greater asset score compared to those with no education. Only amongst women with higher secondary education was there an increase in asset score amongst those with later age at marriage, demonstrating broadly a dose-response increase with marriage age. [file peerj-12-17671-s006.pdf]
